# Modelling and simulation of wood chip combustion in a hot air generator system

**DOI:** 10.1186/s40064-016-2817-x

**Published:** 2016-07-25

**Authors:** J. K. A. T. Rajika, Mahinsasa Narayana

**Affiliations:** Department of Chemical and Process Engineering, University of Moratuwa, Katubedda, 10400 Sri Lanka

**Keywords:** Wood chip, CFD, Modelling, Simulation, Packed bed combustion, OpenFOAM

## Abstract

This study focuses on modelling and simulation of horizontal moving bed/grate wood chip combustor. A standalone finite volume based 2-D steady state Euler–Euler Computational Fluid Dynamics (CFD) model was developed for packed bed combustion. Packed bed combustion of a medium scale biomass combustor, which was retrofitted from wood log to wood chip feeding for Tea drying in Sri Lanka, was evaluated by a CFD simulation study. The model was validated by the experimental results of an industrial biomass combustor for a hot air generation system in tea industry. Open-source CFD tool; OpenFOAM was used to generate CFD model source code for the packed bed combustion and simulated along with an available solver for free board region modelling in the CFD tool. Height of the packed bed is about 20 cm and biomass particles are assumed to be spherical shape with constant surface area to volume ratio. Temperature measurements of the combustor are well agreed with simulation results while gas phase compositions have discrepancies. Combustion efficiency of the validated hot air generator is around 52.2 %.

## Background

Tea industry in Sri Lanka is significant. Ceylon Tea is popular all over the world for its quality since for a long time and Sri Lanka is third largest Tea exporter in the world. In 2013, Tea export has earned about 2 billion US$, which is approximately 14 % of total exports in the country and it is 62 % of total agriculture exports (Sri Lanka Export Development Board 2015). Major contemporary barrier facing in the Tea industry is high energy intensity due to using of inefficient obsolete biomass combustion technologies. In conventional biomass operated systems, 2.4 kg of fuel wood is required to produce 1 kg of “made” tea (Leelaratne 2003) and ninety percent (90 %) of energy requirement is thermal energy and out of that eighty five percent (85 %) comes from biomass which is utilized for drying and withering. Presently various approaches have been implemented to reduce energy consumption for minimizing cost of tea production. Wood log combustors are commonly used in present Tea industries. Then retrofitted wood log combustors by continuous wood chip feeding systems to use wood chips is identified as most viable method to improve the combustion efficiency of existing systems. As wood chips feed from one side of the combustor, it can be considered as a moving packed bed combustion. Then, biomass feeding rate affects to packed bed moving velocity. To understand performance of these systems, it is vital to study thermal fluid flow patterns inside the combustor, which affects to heat transfer to hot air circulations, for temperature controlling of hot air in the tea drying purpose (Rajika and Narayana 2013). In this study, a steady state two-dimensional CFD model was developed for wood chip combustion with moving grate system and a simulation study was accomplished. The CFD model was validated with data from a wood chip combustor, which is used in a Tea factory in Sri Lanka. The CFD model represents the temperature distribution in the packed bed combustor. Although this research focused on a particular combustor, the packed bed combustion model is a generalized model. The whole modelling approach can be used to simulate steady state combustion process. Reactions and thermo-physical properties were evaluated by using existing empirical models, which suits for biomass combustion.

## Literature review

Packed bed combustion models can be primarily categorized in two different approaches. These are according to the directional variations and treatment of the bed particles. Directional variations are considered from zero-dimensional models to three dimensional transient models. One-dimensional transient model is most popular among the pack bed models and it is able to validate macro scale properties like burning rate but not gas compositions (Yang et al. 2002, 2004a; Shin and Choi 2000).

Packed bed solid particles can be considered either as a continuum (macro scale) (Shin and Choi 2000; Ragland et al. 1991; Blasi 1998; Yang et al. 2004b) or discrete particles by using Lagrange particle tracking (particle resolved micro scale) (Mehrabian et al. 2011; Brucha et al. 2003). There are lot of sub-variations by accounting particle shape and size such as cylindrical, spherical, cubic and thermally thin or thermally thick. Apart from those, physical and chemical properties and reaction models are significant for modelling of packed bed combustion. Effective thermal conductivity method is commonly used to simplify different thermal conductivity properties of pack bed materials. The effective thermal conductivity is evaluated by considering combination of thermal conductivities for a quiescent bed with the correction of turbulence created due to mixing. This method is used for both homogeneous and heterogeneous bed models (as separate properties for solid and gas phases). In some models, radiation effect is also lumped into the effective thermal conductivity (Jurena 2012; Sirinivasan 2011), while others were used separate radiation models (Yang et al. 2005a). However, most of the combustion models have used correlations from Wakao and Kaguei (1982) and Bird et al. (2002).

Drying models can be divided into three categories. These are heat sink models, kinetic rate models and equilibrium models. In heat sink models, reaction rate depend only on the heat transfer rate (Wakao and Kaguei 1982). First order kinetic rate models assume drying rate depends only on the particle temperature and moisture content (Jurena 2012). The equilibrium models consider mass and heat transfer both for evaluating drying rate (Zhou et al. 2005). Devolatilization of fuels can be represented by a one-step global reaction (Yang et al. 2005a). Very slow to very fast reaction rates is shown a weak influence to the burning rate but it affects on ignition rate and reaction zone thickness. Char combustion rate is controlled by both diffusion and reaction kinetics, hence it is modelled by considering combination of mass transfer coefficient and reaction kinetic rate constant (Ragland et al. 1991; Mehrabian et al. 2011; Yang et al. 2005a). There is a wide variation of volatile combustion reaction models available in literature. Volatile combustion reaction can be reduced to single reaction by considering major single volatile gas component and neglecting effects of other volatile reactions. Often minimum of mixing rate and kinetic reaction rate are taken as the volatile combustion reaction rate, although there are some instances only kinetic reaction rate is used (Yang et al. 2002, 2005a; Shin and Choi 2000; Wurzenberger et al. 2002).

**Fig. 1:**
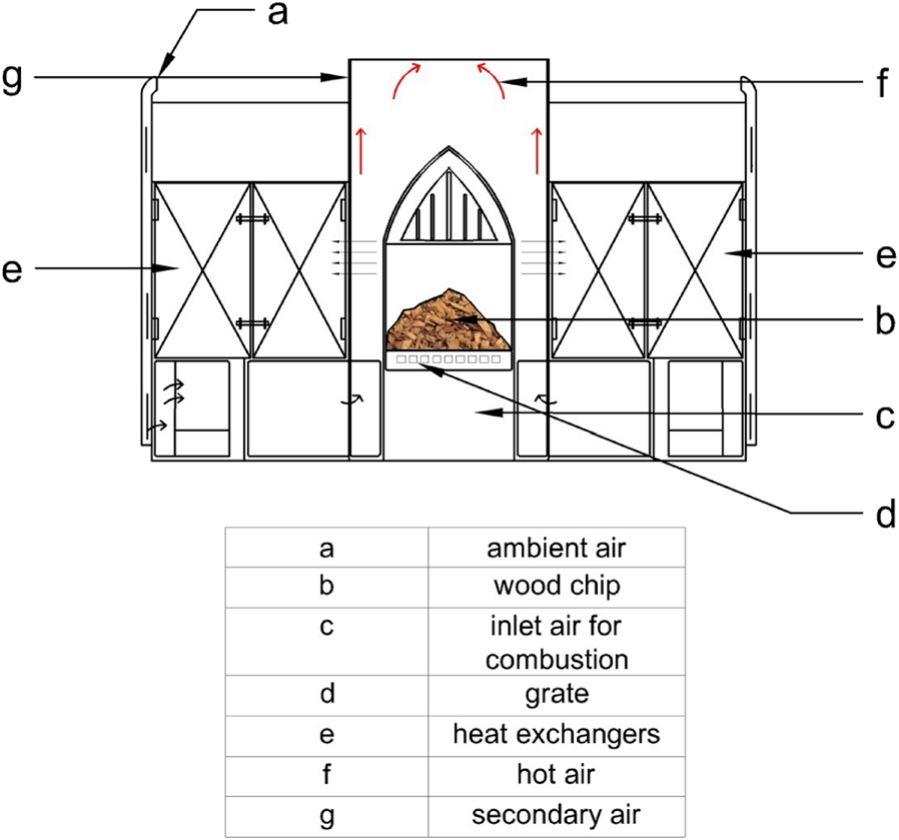
Conventional wood log combustor in tea industry

**Fig. 2:**
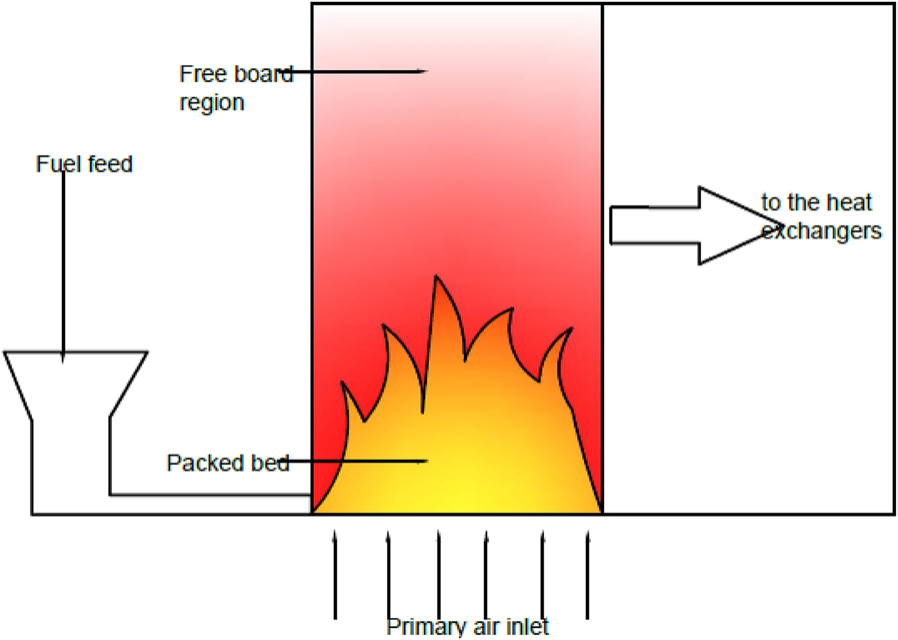
Two-dimensional illustration of hot air generation system (continuous feeding)

The conventional wood log fed combustor system, which is shown in Fig. 1, is used in Sri Lanka. Continuous wood chip feeding is achieved by adding a hopper in front of the grate of combustor. Therefore, this continuous wood chip feeding system can be considered as a horizontal moving bed/grate type. Major components of a Tea drying hot air generating system are the grate combustion chamber and the heat exchanger. The combustion chamber consists of packed bed and free board regions. Graphical illustration of a hot air generator system is shown in Fig. 2. In this study, packed bed and free board were considered as separate CFD models. A model was developed in OpenFOAM for packed bed combustion of wood chips and free board is simulated using reactingFoam solver in OpenFOAM. Two models were combined by incident radiation on the packed bed top surface, which boundary of both models. The free board region generates incident radiation. Then packed bed model was initially simulated by considering an incident radiation. The packed bed top surface temperature value was obtained by the steady state CFD simulation results and then that value can used as boundary conditions to simulate free board region. Incident radiation was evaluated by the CFD simulation of free border region. This process was repeated until temperature value of both packed bed and free board boundary (packed bed top surface) converging to the same value with iterative simulations (Yang et al. 2004a). The packed bed model was developed for thermally thin wood particles, where intra particle temperature gradients are negligible and the thermal Biot number should be less than 0.1 (Bi < 0.1) (Incroprera and Dewitt 2002). Biomass is considered as a composite of moisture, volatile, char and ash. Combustion processes can be divided into 4 sub-processes, which are consisting as follows;

- Moisture evaporation
- Volatile release/char formation
- Combustion of volatiles
- Combustion of char

Biot number is described as:

1 $$Bi = \frac{hL}{\lambda }$$

## Packed bed model assumptions

The two dimensional steady state packed bed combustion model was developed based on following assumptions;

1. The fuel bed is considered as a continuous porous layer consisting of gas and solid phases.
2. Heat of devolatilization reaction is negligible (Jurena 2012).
3. No heat is transferred through the walls of the packed bed (adiabatic process).
4. Packed bed volume does not change with proceeding of combustion but porosity will change (no shrinkage of the bed).
5. Gas flow is incompressible.
6. Pressure drop due to fuel bed resistance to gas flow is included in source terms f_x_, f_y_ and can be approximated by the Ergun’s equation (Ragland et al. 1991).
7. The combustion gas is a mixture of species hydrocarbons and tar represented by H_2_O, O_2_, CO_2_, CO, H_2_, N_2_,CH_4_(light hydrocarbons),C_x_H_y_O_z_ respectively (Jurena 2012).
8. The radiative heat transfer inside the bed can be modelled by effective thermal conductivity.
9. The effects of moving grates can be modelled by the diffusion coefficient and it is considered as a constant (Ragland et al. 1991; Yang et al. 2005b, 2008; Fjellerup et al. 2003; Ferziger and Peric 2012).

## Packed bed CFD model

According to the Assumption 1 in “Packed bed CFD model” section, gas and solid phases are considered as inter-penetrating continuous fluid mediums. Therefore, gas phase is represented by the porosity in a finite volume. Mass, energy and momentum conservation has been separately applied for each phase as shown in next sub sections.

### Gas phase transport equations

Gas phase is presented in a two-dimensional model including species equation for each gas component.

Continuity equation:

2 $$\nabla \cdot \left( {\varepsilon_{b} \rho_{g} V_{g} } \right) = r_{dry} + r_{pyr} + r_{char}$$

Momentum equations:

3 $$\nabla \cdot \left( {\varepsilon_{b} \rho_{g} V_{g} v_{g,x} } \right) = \frac{ - \partial P}{\partial x} + \nabla .\left( {\varepsilon_{b} \mu \nabla v_{g,x} } \right) - f_{x}$$

4 $$\nabla \cdot \left( {\varepsilon_{b} \rho_{g} V_{g} v_{g,y} } \right) = \frac{ - \partial P}{\partial y} + \nabla \cdot \left( {\varepsilon_{b} \mu \nabla v_{g,y} } \right) - f_{y}$$

where:

5 $$f_{x} = \frac{{150\mu v_{g,x} \left( {1 - \varepsilon_{b} } \right)^{2} }}{{d_{p}^{2} \varepsilon_{b}^{3} }} + \frac{{1.75\rho_{g} v_{g,x} \left| {v_{g,x} } \right.}}{{d_{p} \varepsilon_{b}^{3} }}$$

6 $$f_{y} = \frac{{150\mu v_{g,y} \left( {1 - \varepsilon_{b} } \right)^{2} }}{{d_{p}^{2} \varepsilon_{b}^{3} }} + \frac{{1.75\rho_{g} v_{g,y} \left| {v_{g,y} } \right.}}{{d_{p} \varepsilon_{b}^{3} }}$$

Specie conservation equation:

7 $$\nabla \cdot \left( {\varepsilon_{b} \rho_{g} V_{g} Y_{g,i} } \right) = \nabla \cdot \left( {\varepsilon_{b} D_{g,i} \nabla Y_{g,i} } \right) + r_{i} + \varepsilon_{b} \mathop \sum \limits_{j} r_{i,j}$$

Enthalpy equation:

8 $$\nabla \cdot \left( {\varepsilon_{b} \rho_{g} V_{g} h_{g} } \right) = \nabla \cdot \left( {\frac{{\varepsilon_{b} \lambda_{g} }}{{C_{p,g} }}\nabla h_{g} } \right) + \,h_{c} A_{p} \left( {t_{s} - t_{g} } \right) + \left( {r_{dry} + r_{pyr} + r_{char} } \right)h_{s} + \mathop \sum \limits_{j \in G} \Delta h_{j} r_{j}$$

### Solid phase transport equations

Solid phase is moved with grate movement and then solid phase was modelled in one dimension. Moving grate effects are embedded into solid phase mass diffusion coefficient.

Continuity equation:

9 $$\nabla \cdot \left( {\left( {1 - \varepsilon_{b} } \right)\rho_{g} V_{G} } \right) = - r_{dry} - r_{pyr} - r_{char}$$

Specie conservation equation:

10 $$\nabla \cdot \left( {\left( {1 - \varepsilon_{b} } \right)\rho_{g} V_{G} Y_{s,i} } \right) = \nabla \cdot \left( {\left( {1 - \varepsilon_{b} } \right)D_{s,i} \nabla Y_{s,i} } \right) - r_{i}$$

Enthalpy equation:

11 $$\nabla \cdot \left( {\left( {1 - \varepsilon_{b} } \right)\rho_{s} V_{G} h_{s} } \right) = \nabla \cdot \left( {\frac{{\left( {1 - \varepsilon_{b} } \right)\lambda_{s} }}{{C_{p,s} }}\nabla h_{s} } \right) + h_{c} A_{p} \left( {t_{g} - t_{s} } \right) - \left( {r_{dry} + r_{pyr} + r_{char} } \right)h_{s} - r_{c} \Delta h_{c} - r_{dry} \Delta h_{dry}$$

### Gas and solid phase properties modelling

#### Packed bed properties

The packed bed radiation and thermal conductivity effects was lumped into an effective thermal conductivity and this effect was used for the whole bed (Wakao and Kaguei 1982; Jasak 1996). Thermal conductivity is modelled as a combination of effective thermal conductivity for a quiescent packed bed along with a correction for fluid flow which is the method used by most of the researchers in packed bed heat transfer models.

As mentioned in assumption 9 (see “Packed bed model assumptions” section), moving grate effect is modelled by using solid diffusion coefficient, which should be measured practically. In this model, it is considered as a constant (Patankar 1980). Bed porosity is increasing with the advancement of devolatilization and char combustion reactions in solid phase. Pressure drop along the bed and across the bed is depended on air flow resistance of the packed bed particles, which was modelled by using Ergun’s equation.

#### Solid phase reactions

Drying and devolatilization reactions were modelled by considering first order kinetic rate models. The devolatilization product gas components are H_2_, H_2_O, CO, CH_4_, CO_2_ and tar (C_x_H_y_O_z_), which are generated in the ratio of 0.00625:0.3125:0.22875:0.05875:0.14375 and 0.25. Devolatilization rate has been selected from (Yang et al. 2005a) which have found best describes the effects from their previous research works. Char combustion reaction rate cannot be modelled by reaction kinetic rate, since it depends on the oxygen diffusion into the particle surface. Therefore the char combustion rate was modelled by considering both mass diffusion rate and first order kinetic rate (see “Appendix 1”).

#### Gas phase reactions

Volatile gases release from devolatilization oxidization reactions. Reaction rates are shown in the Table 3 in “Appendix 1”. It is assumed that the tar is gaseous specie, which is not condensed and it is combusted similar to paraffin.

## Solution of model equations

OpenFOAM involves co-located grid system and then Rhie–Chow method is used for pressure–velocity coupling (Versteeg and Malalasekara 2007). SIMPLE algorithm, which is used for steady state conditions, is applied to solve the whole system of transport equations, which essentially includes following steps (Karlsson 1995; Karrholm 2006);

1. Approximate the velocity field using momentum equation. Pressure gradient calculated from initial guessing or previous time step values. Equation is under relaxed.
2. Calculation of new pressure filed using pressure Equation. 
3. Calculation of new flux and under-relaxation of pressure and correction of velocity.
4. Solving all other transport equations, under relax equation to improve convergence.
5. Stop if all the convergence criteria are satisfied, else start from 1st step.

The packed bed simulation involves enthalpy equations and species equations of both solid and gas phases. Since most of the model parameters are temperature dependent and convergence criteria of CFD solver have been set as gas phase temperature residuals of 10^−6^.

### Boundary conditions

**Fig. 3:**
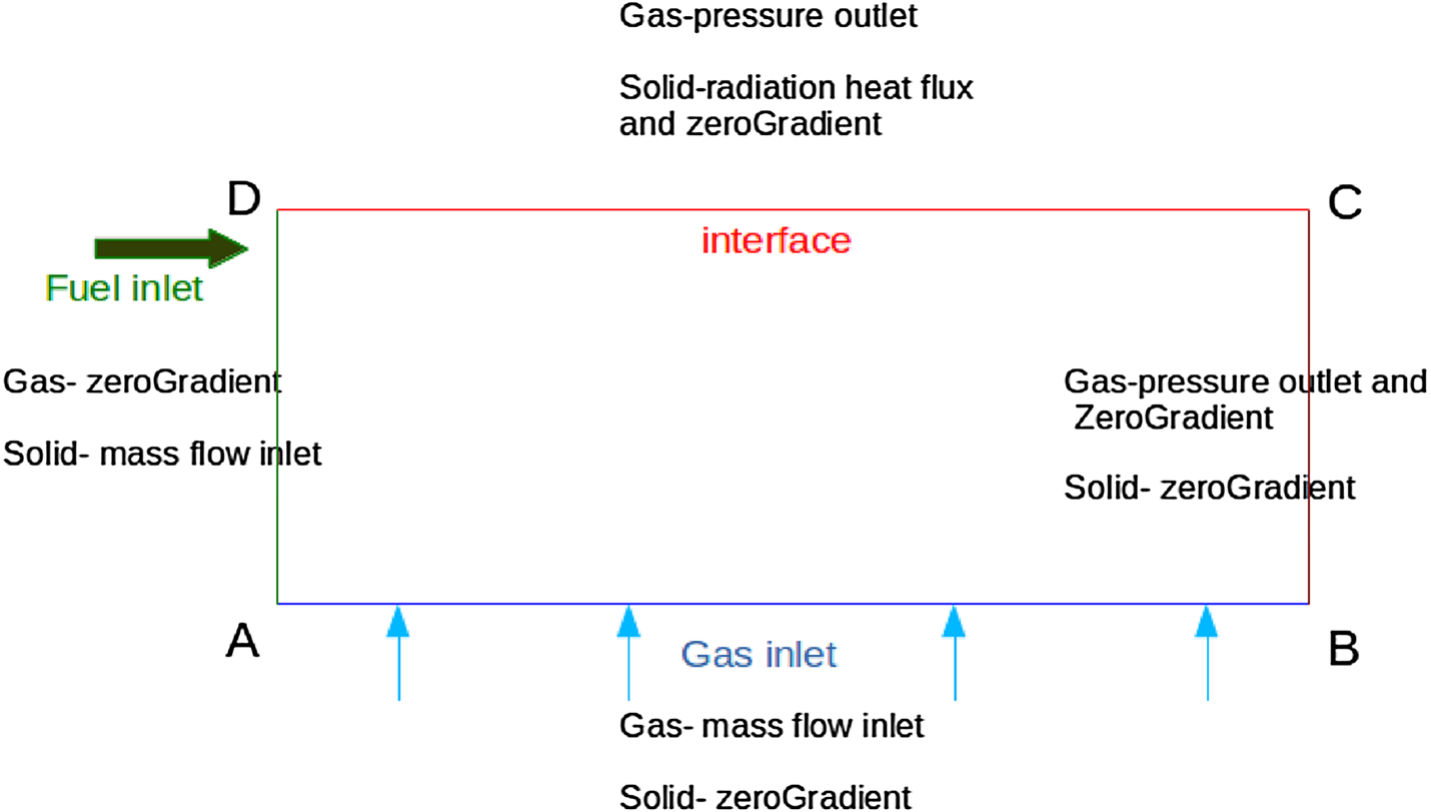
Boundary conditions of the packed bed

**Fig. 4:**
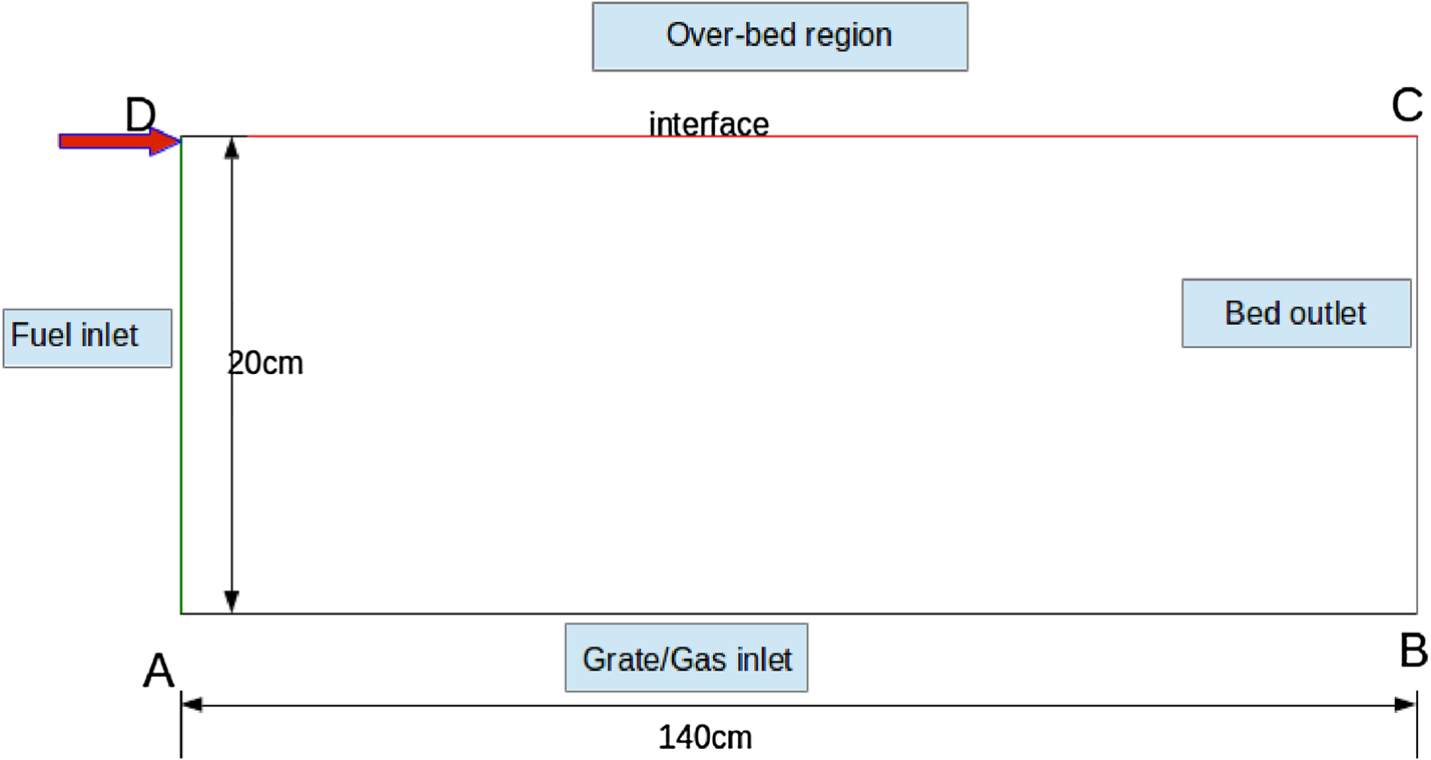
Dimensions of the packed bed

Boundary conditions of the packed bed are shown in the Fig. 3. Dimensions of the packed bed are given in Fig. 4. Hot air generators utilized in Tea industries have fire tube heat exchangers in lateral directions which carry generated hot air using induced draft. Therefore, it is a difficult task to define negative pressure boundary condition (compared to atmospheric pressure 101325 Pa) at BC and AB noted in Fig. 3. It can be assumed that a fixed non-uniform pressure field is increased from B to C respectively. The solid phase temperature of the packed bed at the boundary “interface” is modelled as a radiation heat flux incident on packed bed from free board region. As solid temperature cannot be explicitly added, the radiative heat flux at interface is used to calculate temperature at the interface by following equation (Ragland et al. 1991).

12 $$\left( {\nabla T_{s} } \right)_{interface} = \left( {\varepsilon_{rad} \sigma \frac{{\left( {T_{env}^{4} - T_{top}^{4} } \right)}}{{\left( {1 - \varepsilon_{b} } \right)\lambda_{s} }}} \right)_{interface}$$

This can be further simplified as;

13 $$\frac{{T_{top} - T_{cell} }}{\Delta y/2} = \left( {\varepsilon_{rad} \sigma \frac{{\left( {T_{env}^{4} - T_{top}^{4} } \right)}}{{\left( {1 - \varepsilon_{b} } \right)\lambda_{s} }}} \right)_{interface}$$

where; *T*_*cell*_—temperature of the interface cell, $\Delta y$—height of the interface cell of the bed and *T*_*env*_—radiation temperature.

## Free board region modelling

Free board region, where generated gas components gather from combustion zone, lies just above the packed bed. Therefore, only gas phase presents in this region and turbulence-combustion interaction is occurred. Free board region modelling was carried out by using an available application solver in OpenFOAM called “reactingFoam”. Favre averaged Navier–Stokes equations were used for modelling turbulence and these equations were approximated with Low-Reynold number k − ε model for more accurate results (Perry and Green 1999; Frassoldati et al. 2009). Turbulence-combustion interaction was achieved through Partially Stirred Reactor (PaSR) model in OpenFOAM, which is a model primarily developed for diesel spray combustion and more versatile in contrast of eddy break-up model and laminar flamelet model (Karrholm 2006; Blazek 2001; Rajika and Narayana 2015). Radiation heat transfer was modelled by using finite volume discrete ordinates method. Volatile combustion reactions were modelled by West Brook–Dryer method for oxy-fuel combustion.

Turbulence is transient in nature, but to attain quasi-steady state results to accompany with the steady state packed-bed model neither DNS nor LES models suitable. Therefore, using of RAS model is essential.

## Simulation method

Simulation of the overall system including packed bed and free board region is achieved through the following sequential procedure (Yang et al. 2004a).

1. Assume radiation temperature incident on packed bed (in this model −1125 K).
2. Run the packed bed CFD code on the packed bed geometry.
3. Use the results from packed bed simulation as input conditions to the free board region.
4. Run the free board simulation till approaching a steady state solution.
5. Obtain the radiation temperature incident on packed bed-free board interface from simulation results.
6. Follow steps 2–5 until temperature from packed bed to the free board and incident radiation temperature on the “interface” of packed bed each converging with iterations.

In step 5; radiation temperature from the simulation results have been obtained assuming that the free board gas phase is acting only as a medium of radiation transfer without absorption and scattering.

**Fig. 5:**
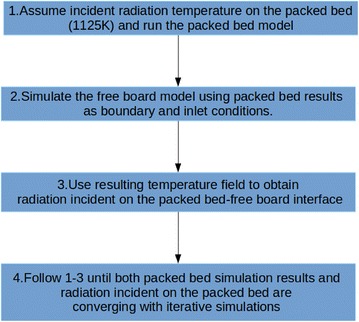
Overall simulation approach for the whole system

Properties in both models which exists, at converging stage are the model results compared with experimental results for validation (Fig. 5).

## Results and discussion

Post-processing of simulation results were carried out with paraview, which is open source visualization third party software along with paraFoam utility in OpenFOAM. Figure 8 in “Appendix 2” shows the convergence of packed bed interface temperature by packed bed simulations. Figure 9 in “Appendix 2” shows radiation temperature incident on packed bed by free board simulations. Packed bed model simulation starts to converge at second iteration and third iteration results have been used as packed bed conditions at steady state.

An experiment was carried out in one of the hot air generators in the Hopewell Tea factory, Balangoda, Sri Lanka, and flue gas temperatures were measured using thermocouples. Gas compositions from free board region simulation have been compared with the flue gas analyzer results.

**Fig. 6:**
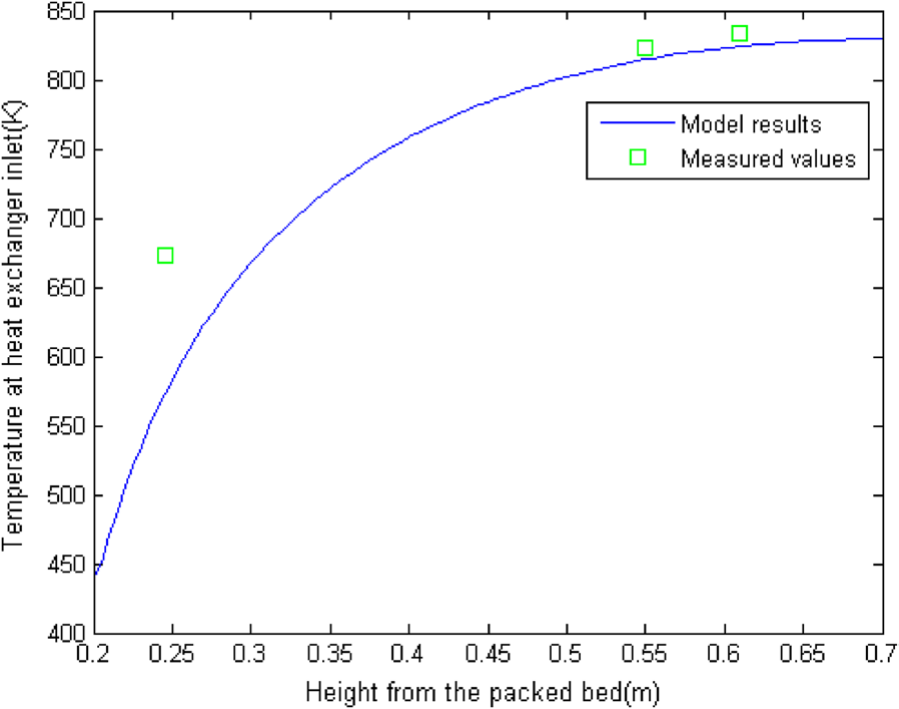
Comparison of temperature at heat exchanger inlet from model and simulation

Temperature measurements are well agreed with simulation results as depicts in Fig. 6, while gas compositions measurements showing significant variations with simulation results.

Development of a CFD model for moving packed bed wood chip combustion is the main object of this study. Modelling and simulation of free board region is preceded by available models for turbulence, chemistry, radiation, etc. in the OpenFOAM. In this study, CFD simulation study was used to evaluate moving bed combustion in a retrofitted wood log combustor by continuous wood chip feeding system, which is used in particular Tea factory in Sri Lanka.

**Fig. 7:**
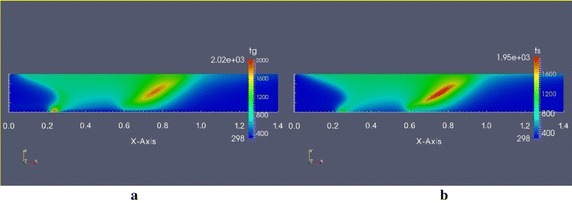
Gas phase and solid phase temperature of packed bed at steady state. **a** Temperature of gas phase (K), **b** temperature of solid phase (K)

**Fig. 8:**
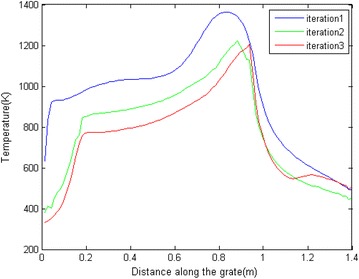
Packed bed gas outlet (interface) temperature with iterations

**Fig. 9:**
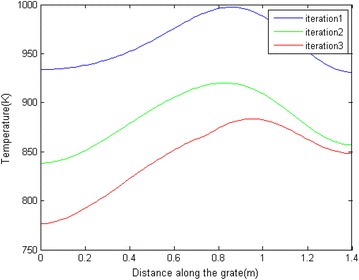
Radiation temperature incident on packed bed

As per the simulation results, high temperature values can be observed in the top of the packed bed due to radiation effect from the free board region. Then drying starts from the top of the bed and progressing downward to the bottom. Reaction occurs in a thin region showing a high rate of reaction compared to char combustion (Fig. 13a in “Appendix 2”). Following the drying, devolatilization reaction occurs in a thin region, which is a rapid reaction compared to char combustion (see the Fig. 13b—“Appendix 2”). According to the CFD simulation, two high temperature regions appear in the packed bed (see Fig. 7). One region is observed at the bottom of the packed bed, which is followed by the drying and devolatilization reaction regions (see Fig. 13a, b—“Appendix 2”). At the same region a high carbon dioxide (CO_2_) generation can be observed (see Fig. 14c “Appendix 2”) without reduction of char mass fractions (see Fig. 13c—“Appendix 2”). Therefore, in this region volatile combustion should be initiated. Further to that this reactive region is moved down along the bed due to lack of oxygen in the upper part of the packed bed. So, in this combustion, gas phase temperature is very high comparatively low temperature in solid phase in the same region. The other high temperature region appears with a co-current reduction of char mass fraction. Therefore, this region should be created due to char combustion. After passing the second high temperature region temperature gradually drops to room temperature by convective cooling of incoming air.

Unlike drying and devolatilization, char combustion starts from the bottom of the bed. It should be due to either lack of oxygen at top or high temperature in the bottom due to volatile combustion.

**Fig. 12:**
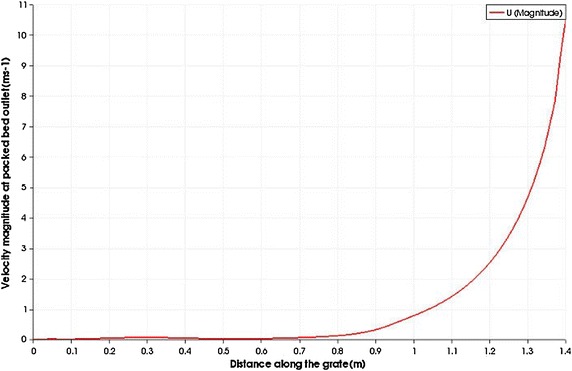
Velocity at packed bed interface along the grate

**Fig. 13:**
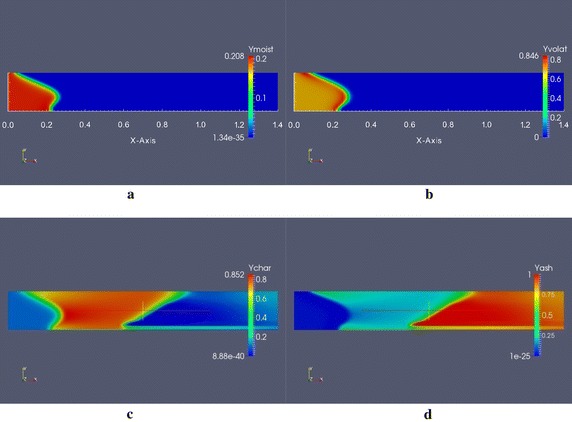
Solid phase component mass fractions. **a** Moisture fraction in solid phase, **b** volatile mass fraction in solid phase, **c** char mass fraction at solid phase, **d** ash mass fractions

**Fig. 14:**
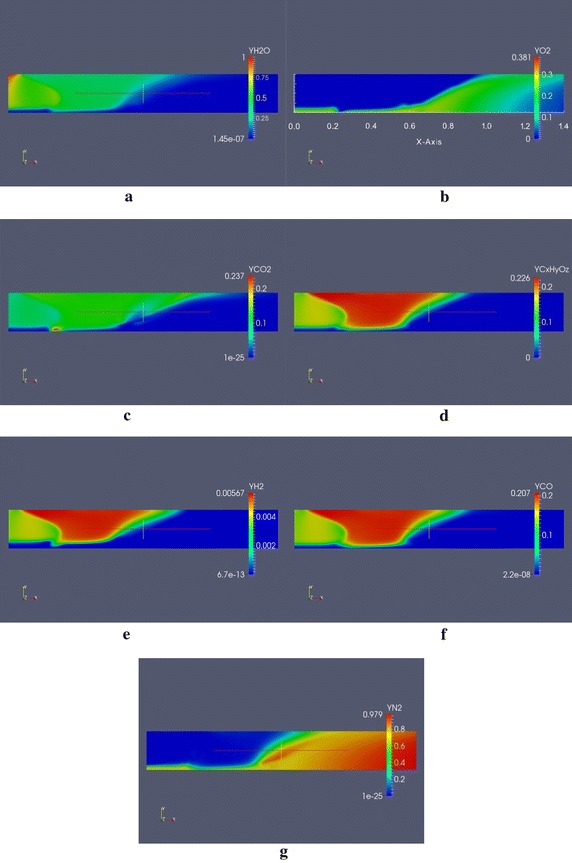
Gas component mass fractions of packed bed at steady state. **a** H_2_O mass fractions, **b** oxygen (O_2_) mass fractions, **c** carbondioxide (CO_2_) mass fractions, **d** C_*x*_H_*y*_O_*z*_ mass fractions, **e** hydrogen (H_2_) mass fractions, **f** carbonmonoxide (CO) mass fractions, **g** nitrogen (N_2_) mass fractions

Char combustion reaction region is thicker than drying and devolatilization reactions region. Therefore, char combustion reaction is implying a slow reaction. Some char is remaining at the bottom of the packed bed without combustion even at the end of the grate. This must be due to the cooling effect of high velocity air flow at the end of the grate. Simulation results illustrate that under the given inputs and boundary conditions of the particular packed bed combustor in the tea factory, insufficient or no air penetration to the freeboard region. This prevents volatile combustion in the freeboard region. The disadvantages are loss of energy and release of harmful gases due to incomplete combustion into the atmosphere such as carbon monoxide (see Figs. 12 and 14g, b of “Appendix 2”).

By the simulation study, following decisions can be made for the particular industrial reactor:

1. Air flow rate is too high at ash pit end of the moving grate. This creates an unnecessary cooling effect.
2. Packed bed outlet (free board region interface) conditions show no excess air in the gas flow.
3. Drying and devolatilization shows rapid reactions with thin reaction zones.
4. Two distinct high temperature regions appear from gas phase volatile combustion and solid phase char combustion reactions.

## Conclusion and recommendations

As illustrated in the discussion, this mathematical model can be used to identify the deficiencies of the packed bed combustion in a particular furnace. The packed bed combustion can be optimized by changing boundary conditions, which depends on the input variables.

CFD modelling and simulation technique for packed bed combustor in Tea industry is an initiative work for critical temperature control of hot air generators. This research work always concerned on developing a simple model for the aforementioned purpose while obtaining accurate results to the required level. The model simulation results were validated by the temperature values of the hot air generator system. Therefore, it can be used for temperature controlling purposes and to predict steady state conditions which are currently a major barrier in Tea drying which highly affects the quality of Tea produced.

 Gas compositions could not predict in an agreed level by the overall model discussed. Therefore, it is necessary to improve the CFD model further, which will be essential to control prospective air pollution. Improving volatile components, volatile reactions in packed bed free board model and volatile reaction rate models shall be considered for further improvements.

## Abbreviations

### List of symbols

A: volumetric particle surface (m^2^/m^3^)/pre-exponential factor (1/s)
Bi: Biot number
C_p_: specific heat capacity (J/kg/K)
c: concentration (kg/m^3^ or mol/m^3^)
D: mass diffusion coefficient (m^2^/s)
d: diameter (m)
h_c_: convective heat transfer coefficient (W/m^2^/K)
h_s_: solid phase sensible enthalpy (J/kg)
h_g_: gas phase sensible enthalpy (J/kg)
L: characteristic length (m)
r: reaction rate (kg/m^3^)
t: temperature (K)
$$\overline{U}$$
time-averaged mean velocity (m/s)
$$v$$
velocity component (m/s)
Y: mass fraction
V: volume (m^3^) or velocity vector (m/s)
*ε*: porosity
*ρ*: density (kg/m^3^)
*μ*: viscosity (kg/m/s)
*λ*: thermal conductivity (W/m/K)

### Subscripts

*ι*: length scale (m)
dry, pyr, char: drying, pyrolysis, char combustion
G: grate
g, s: gas, solid
i, j: ith/jth-component
p: particle
x, y: x/y-direction
0: initial or quiescent

## Figures and Tables

**Fig. 10:**
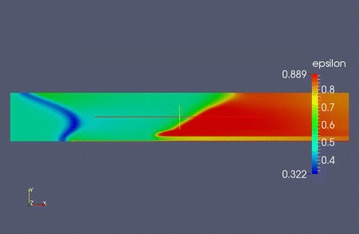
Packed bed porosity at steady state

**Fig. 11:**
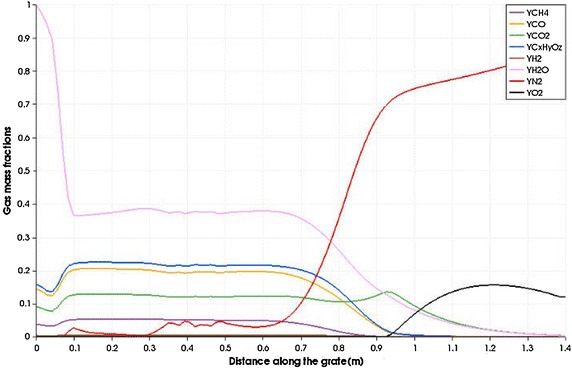
Gas compositions releases to the freeboard

## Appendix 1: Chemical and physical properties modelling

See Tables 1, 2, 3, 4.

**Table 1:** Empirical models used for thermo-physical properties modelling

Property	Model
Gas phase heat capacity (Ragland et al. 1991)	$C_{p,g} \left( {T_{g} } \right) = 990 + 0.122T_{g} - 5680T_{g}$ (J/kg K)
Solid phase heat capacity (Jurena 2012)	$C_{p,wet} = \frac{{C_{p,dry} + 4.19M}}{1 + M} + A$ (kJ/kg K) $A = \left( {0.02355T - 1.32M - 6.191} \right)M$ $C_{p,dry} = 0.1031 + 0.003867T$
Porosity (Wakao and Kaguei 1982)	$\varepsilon_{0} = \frac{{V_{g} }}{V}$ $\varepsilon = \varepsilon_{0} + \left( {1 - \varepsilon_{0} } \right)\mathop \sum \nolimits_{i} f_{i} \left( {Y_{i,0} - Y_{i} } \right)\varepsilon_{0} = 0.5$ i-char, volatile
Gas phase density	1.3 (kg/m^3^)
Solid phase density	500 (kg/m^3^)
Kinematic viscosity	$v = 1.523$
Effective thermal conductivity of the bed (Ragland et al. 1991; Wakao and Kaguei 1982; Jasak 1996)	$\lambda_{e} = \lambda_{e,0} + aPrRe\lambda_{g}$ (W/mK) a = 1 along the bed, 0.5 along bed height
Emissivity	0.9
Gas phase diffusion coefficient (Ragland et al. 1991; Wakao and Kaguei 1982; Jasak 1996; Jasak 1996)	$D_{i,e} = D_{i,0} + ad_{p} \left| {v_{g} } \right|$ (m^2^/s) For temperature < 100 K and P_max_ = 70 atm $D_{AB} = \frac{{\left( {0.0027 - 0.0005M_{AB} } \right)T^{3/2} M_{AB}^{1/2} }}{{P\sigma_{AB}^{2} \varOmega_{D} }}$ $\sigma_{AB} = \frac{{\sigma_{A} + \sigma_{B} }}{q}$ (Å) $\varOmega_{\text{D}} = \left( {44.54T^{0 - 4.909} + 1.911T^{0 - 1.575} } \right)^{0.1}$ $T^{0} = kT/\varepsilon_{AB} \varepsilon_{AB} = \left( {\varepsilon_{A} \varepsilon_{B} } \right)^{1/2}$ $M_{AB} = \left[ {\left( {1/M_{A} } \right) + \left( {1/M_{B} } \right)} \right]^{ - 1}$
Solid phase diffusion coefficient (Patankar 1980)	1.4833 × 10^−6^ (m^2^/s)
Fuel particle properties	
Diameter of particle	Assuming; $\frac{surface\;area}{volume} = 240\left( {\text{constant}} \right)$ $d_{p} = \frac{{6\left( {1 - \varepsilon_{b} } \right)}}{240} = 0.025\left( {1 - \varepsilon_{b} } \right)$ (m)

**Table 2:** Biomass combustion reactions

Reaction	Chemical reaction	Reaction rate (kg/m^3^ s)
Drying	$wet\;biomass \to dry\;biomass + {\text{H}}_{2} {\text{O}}_{\left( g \right)}$	$r_{dry} = 5.6.10^{6} \exp \left( {\frac{ - 10584}{ts}} \right)\left( {1 - \varepsilon_{b} } \right)\rho_{s} Y_{{{\text{H}}_{2} {\text{O}},s}}$
Devolatilization (Yang et al. 2005)	$dry\;biomass \to volatile\;gas + char$	$r_{pr,i} = 7.10^{4} \exp \left( {\frac{ - 9977}{ts}} \right)\left( {1 - \varepsilon_{b} } \right)\rho_{s} Y_{i,s}$
Char combustion (Jurena 2012)	$char + \alpha {\text{O}}_{2} \to 2\left( {1 - \alpha } \right){\text{CO}} + \left( {2\alpha - 1} \right){\text{CO}}_{2}$	$r_{char} = A_{p} \left( {\frac{{{\text{C}}_{{{\text{O}}_{ 2} }} }}{{\frac{1}{{k_{r} }} + \frac{1}{{k_{d} }}}}} \right)$ $k_{r} = 290t_{s} \exp \left( {\frac{ - 10344}{ts}} \right)$ $\frac{\text{CO}}{{{\text{CO}}_{2} }} = 33\;{ \exp }\left( {\frac{ - 4700}{ts}} \right)$

**Table 3:** Volatile combustion reactions

Reaction	Chemical reaction	Reaction rate
Hydrogen combustion	${\text{H}}_{2} + {\text{O}}_{2} \to 2{\text{H}}_{2} {\text{O}}$	$R_{{{\text{H}}_{ 2} }} = 51.8t_{g}^{1.5} { \exp }\left( {\frac{ - 3420}{{t_{g} }}} \right)C_{{{\text{H}}_{ 2} }}^{1.5} C_{{{\text{O}}_{2} }}$
Methane combustion	${\text{CH}}_{4} + \frac{3}{2}{\text{O}}_{2} \to {\text{CO}} + 2{\text{H}}_{2} {\text{O}}$	${\text{R}}_{{{\text{CH}}_{ 4} }} = 1.6 \times 10^{10} \exp \left( {\frac{ - 24157}{{t_{g} }}} \right)C_{{{\text{CH}}_{4} }}^{0.7} C_{{{\text{O}}_{2} }}^{0.8}$
Carbon monoxide combustion	${\text{CH}}_{4} + \frac{3}{2}{\text{O}}_{2} \to {\text{CO}} + 2{\text{H}}_{2} {\text{O}}$	$R_{\text{CO}} = 3.25 \times 10^{7} { \exp }\left( {\frac{ - 15098}{{t_{g} }}} \right)C_{\text{CO}} C_{{{\text{H}}_{2} {\text{O}}}}^{0.5} C_{{{\text{O}}_{2} }}^{0.5}$
Tar combustion	${\text{C}}_{6} {\text{H}}_{6.202} {\text{O}}_{0.2} + 4.4505{\text{O}}_{2} \to 6{\text{CO}} + 3.101{\text{H}}_{2} {\text{O}}$	$R_{{{\text{C}}_{6} {\text{H}}_{6.202} {\text{O}}_{0.2} }} = 1.791 \times 10^{8} t_{g}^{0.5} { \exp }\left( {\frac{ - 20131}{{t_{g} }}} \right)C_{{{\text{C}}_{6} {\text{H}}_{6.202} {\text{O}}_{0.2} }} C_{{{\text{O}}_{2} }}$

**Table 4:** West-Brook Dryer mechanism

Reaction	Reaction rate (cal, mol, l, s)
${\text{CH}}_{4} + \frac{3}{2}{\text{O}}_{2} \to {\text{CO}} + 2{\text{H}}_{2} {\text{O}}$	$5 \times 10^{11} { \exp }\left( {\frac{ - 47800}{RT}} \right)C_{{{\text{CH}}_{4} }}^{0.7} C_{{{\text{O}}_{2} }}^{0.8}$
${\text{CO}} + \frac{1}{2}{\text{O}}_{2} \to {\text{CO}}_{2}$	$2.24 \times 10^{12} { \exp }\left( {\frac{ - 40700}{RT}} \right)C_{\text{CO}} C_{{{\text{H}}_{2} {\text{O}}}}$
${\text{CO}}_{2} \to {\text{CO}} + {\text{O}}_{2}$	$5 \times 8\;{ \exp }\left( {\frac{ - 40700}{RT}} \right)C_{{{\text{CO}}_{2} }}$

## Appendix 2: Simulation results

See Figs. 8, 9, 10, 11, 12, 13, 14.
